# Medication Adherence and its Predictors in Patients With Type 2 Diabetes Mellitus in India: A Systematic Review and Meta‐Analysis of Current Evidence

**DOI:** 10.1002/hsr2.71845

**Published:** 2026-02-17

**Authors:** Saurav Basu, Trina Sengupta, Satyajit Kundu

**Affiliations:** ^1^ Department of Community Medicine ESI PGIMSR & ESIC Medical College and Hospital Kolkata India; ^2^ Public Health, School of Medicine and Dentistry Griffith University Gold Coast Queensland Australia; ^3^ ARCED Foundation Dhaka Bangladesh

**Keywords:** diabetes mellitus, India, medication adherence, meta‐analysis, systematic review

## Abstract

**Background and Aims:**

India has nearly 90 million (10.5%) patients living with type 2 diabetes mellitus (T2DM) with a high burden of suboptimal medication adherence. This systematic review and meta‐analysis aimed to synthesize the determinants of medication adherence in Indian patients with T2DM, estimate the pooled prevalence of adherence, and formulate evidence‐based recommendations for public health policy.

**Methods:**

We conducted a systematic review and meta‐analysis of observational and experimental studies from India, published between January 2020 and December 2024. Electronic databases (PubMed, Scopus, Web of Science) were searched for studies reporting the proportion of patients reporting poor adherence to anti‐diabetic medications.

**Results:**

A total of 534 articles were identified after removing duplicates and assessed for eligibility, of which 16 studies were included in this SRMA. The pooled prevalence of medication non‐adherence among MMAS‐8 using studies (*n* = 4) was 48% (95% CI: 18%–79%), and, overall, among all the studies ranged from 11% to 68%. Predictors of poor adherence included older age, socio‐economic disadvantage, substance use, treatment complexity, psychological distress, polypharmacy, and health‐system access barriers. Information on comorbidity and multimorbidity was sparse. Furthermore, a key limitation was that studies using the Morisky adherence scales (MMAS‐4/8) did not report individual item responses, which precluded a granular analysis of specific reasons for non‐adherence. There were eight studies from South India and six from north and north‐eastern India, indicating a significant lack of geographic diversity.

**Conclusion:**

Medication non‐adherence in patients with T2DM persists as a major public health challenge in India, further exacerbating the country's burgeoning diabetes epidemic. Strengthening primary care can address the primary factors contributing to significant non‐adherence in this population.

## 1 Introduction

Diabetes mellitus (DM) is a premier global public health challenge of the 21st century, defined by the characteristic of chronic hyperglycemia linked to long‐term severe microvascular and macrovascular complications, causing large‐scale disability and death [1]. As per the Global Burden of Disease (2023) estimates, an estimated 529 million people (6.1%) are living with diabetes worldwide, which is projected to increase to 1.31 billion by 2050. Type 2 diabetes mellitus (T2DM) cases represent ≥ 90% of the total diabetes burden, a complex condition driven by a confluence of social, demographic, lifestyle comprising physical activity and diet, environmental, and genetic factors. Low and Middle‐Income Countries (LMICs) bear a disproportionate burden of DM, accounting for an estimated 75% of the global patient population [2, 3].

India has nearly 90 million adults with a prevalence of 10.5% living with T2DM as per a nationally representative survey of the population (2008–2020), representing one in four people with the condition worldwide [4]. The burden of DM in India has accelerated in the past few decades from 26 million cases in 1990 due to an ongoing epidemiological, demographic, social, and nutritional transition, with significantly higher burden in urban compared to rural areas, primarily due to the confluence of poor lifestyle factors, especially sedentarism and unhealthy diet [4, 5]. The escalation of the diabetes epidemic in India has deleterious consequences on public health, expenditure, and economic productivity of the population due to a proportional increase in DALYs [6]. The concomitant high out‐of‐pocket costs for anti‐diabetes medications and hospitalization resulting from disease complications, which distress and risk vulnerable populations sliding into poverty [7, 8].

Achieving and maintaining normoglycemic status with anti‐diabetes medication—either oral drugs or insulin injections is fundamental to preventing long‐term complications. Yet, poor medication adherence remains a major challenge [9]. Beyond terms such as compliance, concordance, fidelity, and persistence, adherence broadly describes the extent to which patients follow instructions for their prescribed treatments. The World Health Organization (WHO) has promoted the term “adherence” for use in chronic disorders as “the extent to which a person's behavior—taking medication, following diet, and/or executing lifestyle changes corresponds with agreed recommendations from a health care provider.” The conception of adherence thereby recognizes the importance of the collaborative nature of the patient provider relationship in maintaining optimal drug behavior Suboptimal medication adherence in individuals with diabetes is a primary driver of emergency department consultations, inpatient admissions, increased morbidity and mortality, and augmented healthcare expenditures [9, 10]. Clinically, medication non‐adherence compromises glycemic control, evident from glycated hemoglobin (HbA1c) levels, which damages blood vessels and accentuates the risk and early onset of developing microvascular and macrovascular complications, including retinopathy, nephropathy, neuropathy, and cardiovascular events, ultimately leading to high rates of morbidity and mortality [11]. Economically, medication non‐adherence amplifies the financial burden of diabetes by increasing healthcare resource utilization, such as emergency room visits and hospitalizations, and elevating the costs associated with managing preventable complications [12]. The magnitude of this problem is such that the WHO has stated that improving adherence to existing therapies would have a far greater impact on public health outcomes than the development of any single new medical treatment [13].

Adherence to long‐term therapies in developing countries is much lower compared to developed countries due to adverse social determinants such as lower levels of patient literacy, and low socioeconomic status resulting in financial constraints and drug unaffordability, while health system barriers undermine healthcare accessibility [10, 14]. Within India, existing evidence reveals an extensive variation in adherence rates and patterns in patients with DM, which may range from as low as 25% in community settings to 75% and higher in facility‐based settings, with further variation attributable to cultural differences in populations across geographic regions, rural and urban variation, and differential health system performance across subnational regions [15, 16]. The heterogeneity is compounded by the non‐use of standardized methodology, differential adherence measurement tools due to a lack of culturally validated tools, misreporting of adherence rate due to misinterpretation of scoring methods, and several medication adherence tools not assessing for financial non‐adherence [17].

Given these significant knowledge gaps, a rigorous synthesis of the available evidence is urgently required to construct a comprehensive, evidence‐based framework that can inform targeted interventions. Therefore, this study was conducted with the objectives of synthesizing and reviewing the determinants of medication adherence in patients with T2DM in India, to estimate the pooled estimation of prevalence of medication adherence in this population, and to provide evidence‐based recommendations for public health research and policy.

## 2 Methods

The protocol for this systematic review and meta‐analysis has been registered in the PROSPERO database (CRD42021236666). The systematic review was conducted in accordance with the PRISMA guidelines (Supporting Information Table S1) [18].

### 2.1 Search Strategy

A systematic search of the PubMed, Scopus, and Web of Science electronic databases was conducted to identify all relevant studies published from January 1, 2020, until December 31, 2024. This period coincides with the dual influence of pandemic‐related disruptions and the systemic reinforcement of India's national program for noncommunicable diseases (2023), which prioritizes the standardization of primary care and medicine accessibility [19, 20].

The search strategy was designed to identify studies focusing on medication adherence in patients with T2DM in India. Search terms included a combination of controlled vocabulary (i.e., Medical Subject Headings [MeSH]) and free‐text keywords covering three main concepts: the population (“Type 2 Diabetes Mellitus,” “T2DM”), the outcome (“medication adherence,” “compliance”), and the geographic location (“India”). The search strategies were adapted for the syntax of each database. The complete and exact search strategies used for all the databases are provided in Supporting Information Table S2.

### 2.2 Inclusion Criteria

We included studies on patients with type 2 diabetes (T2D), with no restrictions on age, gender, or the presence of comorbidities. Exposure was the use of Oral Antidiabetic Drugs (OADs) and insulin. English‐language observational studies (cohort and cross‐sectional), interventional trials with a sample size of more than 50, were included in this SRMA.

### 2.3 Exclusion Criteria

We excluded studies that did not report outcome as adherence (i.e., persistence), and studies not measuring adherence (or non‐adherence) as the number/proportion of adherent (or non‐adherent) patients out of the total Qualitative studies were excluded from this systematic review and meta‐analysis as the review's primary objective is to synthesize quantitative data to determine the overall pooled prevalence of medication adherence in patients with Diabetes in India. We excluded non‐peer‐reviewed gray literature, abstracts, and conference papers (apart from review articles) to maintain methodological rigor and ensure the reliability of the evidence included in this review.

### 2.4 Screening and Selection

A total of 271 PubMed records, 258 Scopus, and 149 Web of Science records were identified, which were imported into Zotero reference management software (Zotero Desktop Version 7), following which the duplicate records were removed. All the titles were then subject to abstract screening in Rayyan software by two authors independently (SB and TS). Then, potentially relevant full texts were retrieved or, when not available, requested from the authors. Two authors (SB and TS) selected eligible studies according to the inclusion and exclusion criteria. Any conflict was resolved through consensus between the authors.

### 2.5 Data Extraction

Information based on the following characteristics has been extracted: the name of the first author, year of publication, name of the journal, study design, study setting, study location, population group, sample size, general characteristics of the participants, like mean age, gender, educational attainment, and so forth, types of co‐morbidity present, and adherence assessment method. The primary outcome measure was the proportion of patients having poor adherence to anti‐diabetic medications. The data extraction process was conducted independently by two authors. Data were collected with the spreadsheet software Microsoft Excel 365.

### 2.6 Risk of Bias (RoB) Assessment

The RoB of the included studies was evaluated with a modified version of the Joanna Briggs Institute appraisal checklist for studies reporting on prevalence data [21]. The tool is composed of 8 domains that consider inclusion criteria, a detailed description of the study subjects and setting, valid measurement of exposure, statement of objectives and standard criteria, confounding factors, strategies to deal with confounders, valid and reliable measures of the outcomes, and use of appropriate statistical analysis. Each domain is measured using four types of responses: Yes/No/Unclear/Not Applicable. Since the JBI tool does not provide a scoring system, we adopted a binary scoring approach, assigning a score of “1” for responses marked “Yes” and “0” for responses marked “No” or “Unclear.” When an item was deemed “Not Applicable,” it was excluded from the denominator for that particular study. This allowed us to calculate a percentage score reflecting the overall quality of each study. Evidence certainty was also appraised via the GRADE approach [22].

### 2.7 Statistical Analysis

The extracted data were entered in Microsoft Excel, and all statistical analyzes were performed using R statistical software (version 4.5.1). The meta‐analysis was conducted using the metafor package (version 4.8.0) and the meta package (version 8.1.0) for calculating effect sizes, pooling results, and assessing heterogeneity and publication bias. Other packages used for data manipulation and visualization included dplyr (version 1.1.4) and ggplot2 (version 3.5.2). Inconsistency index (I^2^) was used to report statistical heterogeneity. The I^2^ values of 25%, 50%, and 75% are regarded as low, moderate, and high heterogeneity, respectively. Since there was significant heterogeneity between the studies, the random effects model was used to calculate the pooled estimates for measuring adherence to anti‐diabetic medications. The pooled estimate was expressed as proportions with 95% confidence intervals (CI). Publication bias was assessed using a funnel plot visualization, with the presence of small‐study effects statistically tested through Egger's regression test, setting the significance threshold at *p* < 0.05.

## 3 Results

### 3.1 Identification of Studies

**Figure 1:**
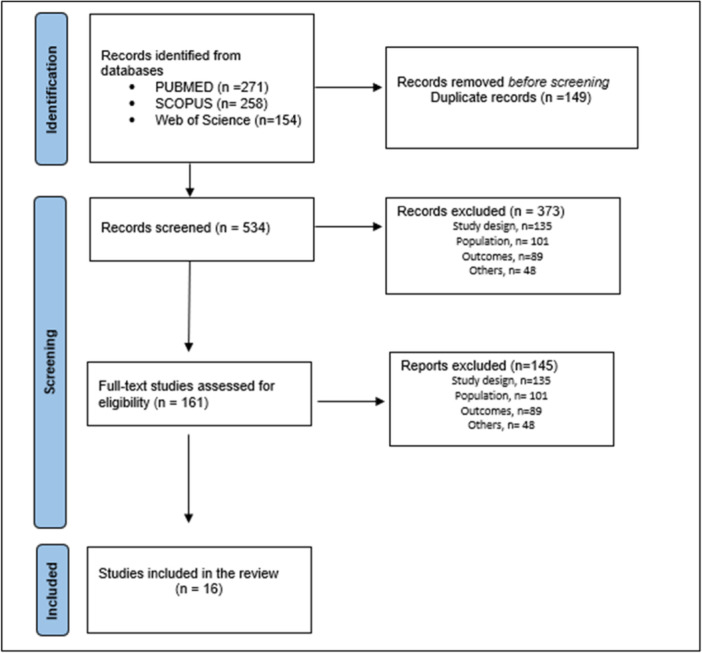
PRISMA flow diagram of the systematic review and meta‐analysis.

A total of 534 records were initially identified, meeting the inclusion criteria on title cum abstract screening. After removing duplication, a total of 161 full texts were assessed for eligibility, of which 16 studies were finally selected for the systematic review [23, 24, 25, 26, 27, 28, 29, 30, 31, 32, 33, 34, 35, 36, 37, 38]. (Figure 1).

### 3.2 Characteristics of Included Studies

Of the included studies, 14 were cross‐sectional [23, 24, 25, 26, 27, 28, 29, 30, 31, 32, 33, 36, 37], one was prospective longitudinal [38], and one utilized a mixed‐methods convergent parallel design [34]. Only four studies were conducted at the community level [23, 25, 26, 27], and the rest were facility‐based [24, 28, 29, 30, 31, 32, 33, 34, 35, 36, 37, 38]. The average sample size of the studies was 260.62. The mean (SD) age of the participants in the included studies was 55.70 (11.01) years, and about 46.7% were men. As per the geographical location, 8 studies were conducted in the North and North‐Eastern India, and 8 were from South India (Table 1).

**Table 1:** Characteristics of the included studies.

Author	Year of publication	Location	Effective sample size	Study design	Study setting	Study population	Mean (SD) age	Men/Women	Literacy
Aravindakshan et al. [23]	2021	Kerala	218	Cross‐sectional	Community based	≥ 30 years	62.13 (12.22)	131/87	1.3% were illiterate,
Ghosh et al. [24]	2022	West Bengal	165	Cross‐sectional	OPD based	≥ 30 years	53.90 (11.06)	100/65	16.9% primary education
Hulugappa et al. [25]	2022	Karnataka	70	Cross‐sectional	Community based	≥ 18 years	—	—	—
Kowsalyal [26]	2020	Tamil Nadu	60	Cross‐sectional	Community based	≥ 18 years	—	32/28	57% non‐formal education
Lyngdodh et al. [27]	2021	Manipur	250	Cross‐sectional	Community based	≥ 30 years	46.96 (13.78)	154/96	10.8% illiterate
Mishra et al. [28]	2023	Odissa	207	Cross‐sectional	OPD based	≥ 18 years	54.29 (9.51)	83/124	29.50% illiterate
Mishra et al. [29]	2021	Uttarakhand	277	Cross‐sectional	Hospital based	21–75 years	50.80 (10.6)	158/119	18.4% were illiterate, 91 had primary school education
Olickal et. al. [30]	2021	Tamil Nadu	1002	Cross‐sectional	Hospital based	≥ 18 years	—	616/386	20.9% had no formal education
Prathap et. al [31]	2021	Tamil Nadu	150	Cross‐sectional	OPD based	≥ 18 years	—	83/67	—
Sahoo et al. [32]	2022	Odissa	331	Cross‐sectional	Hospital based	≥ 18 years	53.40 (11)	189/142	19% up to high school
Sharma et al. [33]	2023	Chandigarh	400	Cross‐sectional	OPD based	≥ 18 years	54.6 (11.5)	185/	17% were illiterate
Sharma et al. [34]	2020	Uttarakhand	225	Exploratory study with convergent parallel design	OPD based	T2DM patients who were prescribed insulin for the first time	54.9 (12.9)	109/116	39.1% illiterate
Singh et al. [35]	2021	North India	350	Cross‐sectional	OPD based	≥ 18 years	—	180/170	—
Thapar et al. [36]	2020	Karnataka	124	Cross‐sectional	Hospital based	≥ 18 years	59.8 (11.2)	68/56	—
Udupa et al. [37]	2023	Karnataka	101	Cross‐sectional	Hospital based	> 60 years	66.14 (5.81)	72/29	25.7% illiterate
Verma et al. [38]	2024	Maharashtra	240	Prospective, Longitudinal	OPD based	≥ 18 years		125/115	2.5% illiterate

### 3.3 Adherence to Anti‐Diabetic Medications

The prevalence of poor medication adherence varied considerably across the 16 included studies. Notably, only four studies, both cross‐sectional and based in outpatient departments in Northern and Eastern India, reported poor adherence in more than 50% of their participants [24, 29, 32, 35]. Medication adherence was assessed through self‐report and multiple medication adherence assessment questionnaire scales. The Morisky Medication Adherence Scale (MMAS‐8 or MMAS‐4) was the most common, employed by five studies [24, 26, 32, 36, 38]. Three studies utilized the Medication Adherence Rating Scale (MARS) [29, 31, 35], and one study each employed the Morisky‐Green‐Levin (MGL) scale [30], Brief Medication Questionnaire [33], the Hill‐Bone Medication Adherence Scale (HB‐MAS) [28], and the Summary of Diabetes Self Care Activities Scale [27]. Three studies utilized self‐designed questionnaires for assessing medication adherence that were validated using content validity methods [23, 25, 35]. The scope of adherence assessment also differed: nine studies analyzed adherence to both insulin and OADs, six focused solely on OADs. One study (Sharma S et al.) specifically evaluated insulin adherence using the Barriers to Insulin Treatment Questionnaire, reporting a 46.67% rate of poor adherence. This exploratory, mixed‐methods study conducted in Northern India identified several key barriers through qualitative interviews and focus groups, including low self‐efficacy, skepticism regarding clinical benefits, fear of hypoglycemia, and needle phobia [34]. Another study by Verma et al. investigated interventions for improving medication adherence in patients with DM. The researchers applied mobile telephony (SMS) and pillbox interventions to two groups of patients with poor adherence. After 3 months post‐intervention, both groups demonstrated significant improvements in both their medication adherence and glycemic control [36]. A total of four studies were conducted in community settings, among which medication nonadherence ranged from 22% to 38% [23, 25, 26, 27]. Another 12 studies were conducted in outpatient or hospital settings, where the rates of adherence to anti‐diabetic medications ranged from 20.56% to 68% (Table 2).

**Table 2:** Adherence to anti‐diabetic medications in the included studies from India (2020–25).

Author	Assessment methods	Adherence assessed for	Co‐morbidity	Adherence definition	Prevalence of medication non‐adherence
Aravindakshan et al. [23]	Content validated questionnaire (8 items) based on literature review	OHA	Not reported	1–4 poor adherence, 4.1–7.9 moderate, 8 good adherence.	0.11
Ghosh et al. [24]	MMAS‐8#	OHA	Not reported	Score < 6/8 poor adherence	0.60
Hulugappa et al. [25]	Pretested questionnaire containing 8 questions	OHA, Insulin	50% had comorbidities. Hypertension (94.2%), Asthma (2.9%), and Thyroid 1 (2.9%).	0–2 good adherence, 3–8 poor adherence	0.38
Kowsalyal [26]	MMAS‐8	OHA	Not reported	Not reported	0.22
Lyngdoh et al. [27]	Summary of Diabetes Self‐Care Activities Measure	—	Obesity, hypertension	Not reported	0.15
Mishra et al. [28]	Hill‐Bone Medication Adherence Scale (HB‐MAS)	OHA, Insulin	Not reported	< 80% score poor adherence	0.38
Mishra et al. [29]	MARS* and Section II	OHA, Insulin	thyroid (54.5%) and hypertension (41.5%).	0–5 poor adherence 6–10 good adherence	0.56
Olickal et al. [30]	Morisky Green Levine Adherence Scale	OHA, Insulin	Hypertension, arthritis, asthma, etc.	≥ 1 poor adherence 0 good adherence	0.39
Prathap et al. [31]	MARS 5	OHA	Not reported	Not reported	0.40
Sahoo et al. [32]	MMAS‐8	OHA, Insulin	hypertension (45.6%), arthritis (9.7%), asthma/other chronic respiratory diseases (7.9%), chronic kidney disease (2.1%), and cardiovascular diseases (0.6%)	Not reported	0.65
Sharma et al. [33]	Brief Medication Questionnaire (BMQ)	OHA	present in 54.8%	≥ 1 poor adherence 0 good adherence	0.21
Sharma et al. [34]	The Barriers to Insulin Treatment Questionnaire	Insulin	Not reported	—	0.47
Singh et al. [35]	13‐item questionnaire	OHA, Insulin	Not reported	13 high adherence < 13 low adherence	0.68
Thapar et al. [36]	MMAS‐8	—	Not reported	Not reported	0.43
Udupa et al. [37]	MARS	OHA	Not reported	0–3 Non‐Adherent, 4–6 Partially Adherent 7–10 Adherent	0.28
Verma et al. [38]	MMAS‐4	OHA, Insulin	Hypertension (23%), hypothyroidism (4%), neuropathy (11%)	≥ 1 poor adherence	0.46

* **MARS:** Medication Adherence Rating Scale.

^#^ **MMAS:** Morisky Medication Adherence Scale.

### 3.4 Heterogeneity

Substantial heterogeneity was observed across the studies using the “meta” package, with an estimated τ2 of 0.0349 [0.0183; 0.0845], corresponding to a τ of 0.1868 [0.1354; 0.2907]. The I^2^ statistic, indicating the percentage of total variation across studies due to heterogeneity rather than chance, was 97.3% (95% CI: [96.5%; 97.9%]). A formal test for heterogeneity (Cochran's Q) yielded a Q‐statistic of 551.50 with 15 degrees of freedom, resulting in a *p*‐value of < 0.0001, further confirming significant heterogeneity. Again, the 95% prediction interval (PI) ranged from 10.7% to 76.5%, indicating that the prevalence in a new study conducted in a similar population is expected to lie between 10.7% and 76.5%, considering between‐study heterogeneity.

### 3.5 Pooled Prevalence of Medication Adherence

**Figure 2:**
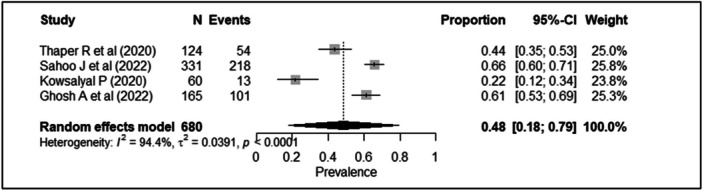
Forest plot showing meta‐analysis of prevalence of non‐adherence to antidiabetic medications (4 studies that utilized the 8‐item Morisky Medication Adherence Scale).

Four studies utilized the 8‐item Morisky Medication Adherence Scale (MMAS‐8) to assess adherence (*n* = 920) [24, 26, 32, 36]. A random‐effects meta‐analysis revealed a pooled prevalence of poor adherence of 48% (95% CI: 18%–79%). Considerable heterogeneity was observed (τ² = 0.039; τ = 0.19), with an I² of 94.4% (95% CI: 87.7%–97.2%) and H = 4.22 (95% CI: 2.98–5.98). Cochran's Q test indicated statistically significant heterogeneity (Q = 53.45, df = 3, *p* < 0.0001). The individual study estimates and the pooled proportion for the MMAS subgroup are presented in the forest plot (Figure 2).

**Figure 3:**
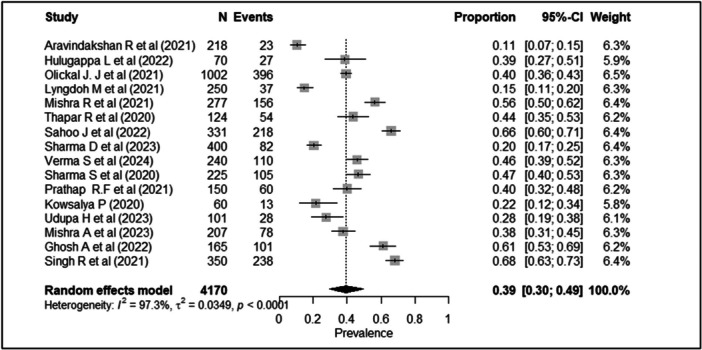
Forest plot showing sub‐group meta‐analysis of prevalence of non‐adherence to antidiabetic medications (all 16 studies).

When including all 16 studies (*n* = 4170) from the systematic review in the meta‐analysis, medication adherence ranged from 11% to 68%. The pooled prevalence of medication nonadherence with this method employing a random‐effects model was 39.3% (95% CI: 29.6%–49.4%) (Figure 3). Based on the GRADE criteria, the certainty of this evidence was rated as very low. This rating was driven by serious concerns regarding the risk of bias in primary studies, very serious inconsistency due to heterogeneity in operational definitions and methods in estimating adherence (I^2^ = 97.3%), with substantial evidence of geographic indirectness and publication bias (Table 3). The forest plot in Figure 3 illustrates the prevalence estimates from individual studies and the overall pooled estimate.

**Table 3:** GRADE summary of findings for medication adherence in patients with type 2 diabetes mellitus in India.

Outcome	Anticipated absolute effect (95% CI)	No. of participants (studies)	Study design	Risk of bias	Inconsistency	Indirectness	Imprecision	Publication bias	Certainty of evidence (GRADE)
Prevalence of medication adherence	39.3% (29.6%–49.4%)	4170 (16 studies)	Observational (cross‐sectional, cohort)	Serious¹	Very serious²	Serious³	Not serious	Serious⁴	Very low

^1^ **Risk of bias:** Several included studies were of moderate to low methodological quality, with limited adjustment for confounding, reliance on self‐reported adherence measures, and potential social desirability bias.

^2^ **Inconsistency:** Substantial unexplained heterogeneity was observed (I2 = 97.3%), with wide prediction intervals reflecting marked variability. This inconsistency is likely driven by methodological factors, including the use of small sample sizes and the lack of standardized adherence cut‐offs (or non‐reporting thereof), which led to unstable estimates and diverse measurement thresholds across the included studies.

^3^ **Indirectness:** Evidence was derived predominantly from facility‐based studies and limited geographic regions, with under‐representation of community settings and several Indian regions, limiting generalizability.

^4^ **Publication bias:** Funnel plot asymmetry and statistically significant Egger's regression test (*p* = 0.027) suggested small‐study effects and potential publication bias.

### 3.6 Predictors of Poor Adherence to Anti‐Diabetic Medications and Pooled Odds

Owing to substantial clinical and methodological heterogeneity across studies, particularly in outcome definitions, exposure categorization, and effect measures (OR, AOR, APR), statistical meta‐analysis was not appropriate. Therefore, a SWiM‐guided vote‐counting synthesis based on the direction of effect was conducted [39]. As summarized in Table 4, across eight studies, older age, socio‐economic disadvantage, substance use, treatment complexity, psychological distress, and health‐system access barriers were consistently associated with medication non‐adherence. In contrast, regular physical activity, social insurance coverage, absence of side effects, and better disease‐related knowledge were associated with improved adherence. Most predictors were reported by single studies, and findings should therefore be interpreted cautiously.

**Table 4:** Effect‐size estimates (OR/AOR) of predictors of medication adherence across included studies (8 out of 16 studies)*.

Study*	Predictor	Effect measure	Estimate	Confidence interval	Sample size
Aravindakshan et al. [23]	Age	OR	6.13	2.25–17.73	218
	Socio‐economic status	OR	0.2	0.05–0.71	
Hulugappa et al. [25]	Sometimes forget to take medication	OR	25.16	6.78–93.28	70
Mishra et al. [28]	Having social insurance	AOR	2.73	1.01–7.85	207
	Current smoker	AOR	5.47	1.56–19.24	
	Anxiety	AOR	3.52	1.62–7.61	
	Regular physical activity	AOR	0.311	0.12–0.79	
Olickal et al. [30]	Female sex	APR	1.44	1.19–1.74	1002
	Daily wager	APR	1.70	1.14–2.54	
	Tobacco user	APR	1.46	1.16–1.84	
	> Distance health facility	APR	1.21	1.04–1.41	
Thapar et al. [32]	Absence of side effects	AOR	2.18	1.02–4.61	124
Sahoo et al. [33]	Presence of comorbidity	AOR	3.26	1.93–5.5	331
	Family history	AOR	1.885	1.11–3.17	
	Alcohol consumption	AOR	2.357	1.03–5.36	
Sharma et al. [34]	Age	OR	1.8	1.1–2.9	400
	Duration of DM	OR	1.8	1–3.2	
	Knowledge	OR	1.8	1.1–3.1	
Verma et al. [38]	Polypharmacy	OR	3.15	1.73–5.75	240
	Taking injectable drugs	OR	0.23	0.09–0.06	

* Studies that reported associations between non‐adherence and various factors.

### 3.7 Publication Bias

**Figure 4:**
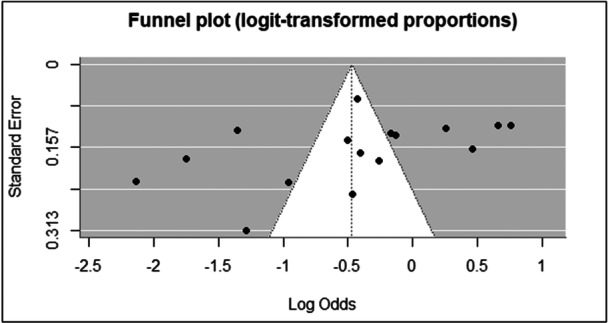
Funnel plot showing publication bias.

A funnel plot was generated to assess potential publication bias (Figure 4). Visual inspection of the plot revealed asymmetry, suggestive of the presence of publication bias; although this should be interpreted cautiously due to the high level of heterogeneity (I² = 97.3%), which can also be a cause of funnel plot asymmetry. To evaluate funnel plot asymmetry, we performed Egger's linear regression test. The mixed‐effects meta‐regression model revealed statistically significant asymmetry (z = −2.205, *p* = 0.027), indicating that the distribution of effect sizes is skewed, likely due to publication bias. This finding suggests that smaller studies tend to report more favorable or extreme effect sizes, which is a common characteristic of publication bias. The limit estimate (intercept) of the regression model, representing the expected effect size for a study with zero standard error, was b = 0.6776 (95% CI: – 0.4032, 1.7583). Despite the significant test for asymmetry, the wide confidence interval around the intercept indicates uncertainty regarding the magnitude of the bias or the true underlying effect size.

### 3.8 Risk of Bias Assessment

Two authors independently assessed the methodological quality of the included studies. The RoB of the included studies was evaluated with a modified version of the tool for observational studies developed by the Joanna Briggs Institute. The total quality scores ranged from 42.86% to 85.71%, with eight studies achieving scores ≥ 70%, indicating moderate to high methodological rigor. Based on the percentage scores, we categorized study quality as high (≥ 75%), moderate (50%–74%), and low (< 50%). Out of the 16 included studies, eight studies (50%) were rated as high quality [24, 25, 29, 30, 32, 34, 36, 38], six studies (35.7%) were of moderate quality [23, 29, 31, 33, 35, 37], and two studies (14.3%) were rated as low quality [26, 27]. Common areas of methodological weakness included limited identification and management of potential confounding factors and incomplete reporting of outcome measurement validity. Despite these variations, the majority of studies demonstrated clearly defined inclusion criteria and adequately described study settings (Supporting Information Table S3).

**Figure 5:**
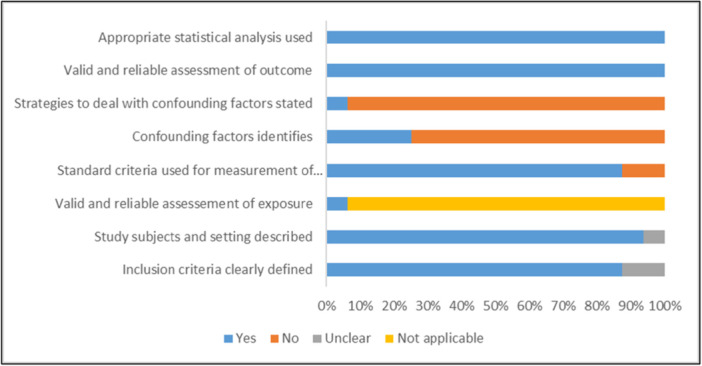
Methodological quality of included studies.

While the overall quality of the studies was satisfactory, a significant limitation was the inadequate identification and handling of confounding factors. Only half of the studies reported factors associated with medication non‐adherence through bivariate or multivariate analysis (Table 4). Most studies failed to assess social desirability bias (SDB), a type of response bias where survey respondents respond in a way they believe will be viewed favorably by others. Researchers generally agree that SDB likely leads to overestimations of medication adherence rates when based on patient self‐reports. Notably, only two studies suspected the presence of SDB in their participants' adherence responses [27, 29]. Additionally, none of the studies utilizing any of the Morisky adherence scales reported item‐wise responses and instead only reported the pooled scale scores, which precluded identifying the reasons that maximally contributed to nonadherence in the respondents (forgetfulness, carelessness, fear of side effects, feeling better, etc.) Certain important risk factors of non‐adherence, such as depression and disability, and protective factors, such as family support, were not assessed in any of the studies included in this SRMA [40, 41]. Similarly, the extent of patient knowledge about diabetes, which may improve adherence and drug persistence, was not formally measured using validated instruments in any of the studies (Figure 5) [42].

### 3.9 Sensitivity Analysis

The leave‐one‐out analysis and Baujat plot reveal important insights into the robustness and heterogeneity of the meta‐analysis results. Leave‐one‐out sensitivity analysis shows that omission of any individual study does not materially alter the pooled prevalence (range: 0.39–0.44) nor reduce heterogeneity (I²: 95.9%–96.8%) (Supporting Information Figure S1). The Baujat plot identifies studies contributing disproportionately to heterogeneity. Singh et al. (2021) shows the highest combined influence on heterogeneity and pooled effect, followed by Sharma et al. (2023) and Sahoo et al. (2022), while most studies contribute minimally (Supporting Information Figure S2). The leave‐one‐out (LOO) influence analysis evaluates how the pooled prevalence changes when each study is removed one at a time. This figure shows that removing any single study does not materially change the pooled effect, as all recalculated estimates remain close to the overall pooled prevalence (around ~ 0.40). Although a few studies, particularly Aravindakshan et al. (2021), Lyngdoh et al. (2021), and Sharma et al. (2023), shift the pooled estimate slightly upward when removed, these variations are minor and remain within overlapping confidence intervals. No study produces a substantial deviation or changes the direction or magnitude of the effect (Supporting Information Figure S3).

## 4 Discussion

This meta‐analysis among patients with T2DM in India reveals concerning low levels of medication adherence, representing a major barrier to achieving optimal glycemic control and stable long‐term health outcomes in the absence of complications. Furthermore, the fragmented nature of current research on adherence in India, characterized by a lack of standardized reporting, small sample sizes, geographically restricted, and a lack of longitudinal data, precludes robust synthesis of drivers of non‐adherence and entails significant gaps in understanding.

Another SRMA among patients with DM in LMIC countries reported the pooled estimate of medication nonadherence in studies utilizing the MMAS‐8 as 43.4% (95% CI: 17.5–69.4) and those utilizing the MMAS‐4 as 29.5% (95% CI: 25.5–33), suggesting comparatively overall higher rates of adherence compared to the Indian population [43].

While this meta‐analysis identified a significant difference in adherence rates between community and facility‐based settings, the magnitude of this disparity has narrowed considerably compared to findings from a decade ago. For instance, studies in Kerala (2013) and Delhi (2015) previously reported a three‐fold difference in adherence (measured by MMAS‐8 among DM patients), suggesting improved medication access in recent years, likely due to the strengthening of primary healthcare facilities [15, 16]. The transition of primary health sub‐centers into Health and Wellness Centers—a key mandate of the national health agenda is intended to optimize the continuum of care for NCDs by guaranteeing uninterrupted access to standard care, including essential medications and diagnostic services in low‐resource settings, particularly in rural India [44, 45]. However, while national health facility assessment surveys indicate enhanced medication availability at all levels of Indian healthcare facilities, challenges persist in achieving supply chain resilience in ensuring uniform accessibility to anti‐diabetic medications without stockouts [46, 47].

In this SRMA, factors contributing to non‐adherence were primarily related to patient‐centered factors (male gender, smoking or alcohol addiction, poor knowledge of diabetes), low socioeconomic status, disease factors (presence of comorbidities), and therapy‐related factors (insulin or OADs). However, half of the studies did not report on the predictors of medication nonadherence, and also failed to identify, the key determinants of nonadherence as per the WHO framework, especially those pertaining to disease‐related dimensions (e.g., duration, complications) and critical health‐system factors, such as irregular medication supply or insufficient health promotion efforts by healthcare professionals in outpatient and community settings [10]. Furthermore, most studies did not report the disaggregated results of the medication adherence scale items that measure factors driving non‐adherence, such as forgetfulness, carelessness, fear of side effects, etc., an avoidable limitation which should be corrected in future studies. Another important limitation was that none of the studies assessed medication non‐adherence in the other comorbid conditions in patients with diabetes related multimorbidity, which can undermine the patient's ability to adhere to their treatment plan. A complex interplay of factors, including polypharmacy, adverse drug effects, and dosing errors due to low self‐efficacy, can significantly compromise medication adherence in patients with T2DM, often leading to poor therapeutic outcomes and a reduced quality of life [48, 49].

Understanding medication adherence requires acknowledging its dynamic nature, as it is not a static behavior often influenced by the presence of other comorbidities, mental health, and access to treatment [50, 51]. Unfortunately, the absence of prospective designs among the observational studies in this review meant that the long‐term trajectory and variability of medication adherence could not be adequately captured or analyzed. Given the varying rates of medication non‐adherence in diabetes, healthcare providers must give greater attention to how multimorbidity affects patients' ability to self‐manage their chronic condition.

Most studies included in this review lacked qualitative insights or a mixed‐methods approach, which limited their ability to explore in‐depth patient perspectives on medication non‐adherence. Consequently, unlike qualitative research, these quantitative studies often failed to identify critical barriers such as patients' lack of understanding about diabetes, fear of complications, and insufficient family support. Crucially, the facilitators for medication adherence, such as the availability of reminder systems, improved awareness of their condition, trust in healthcare providers, and peer support, were not assessed or recognized in any of the studies [52]. They also missed nuanced health system‐related barriers, including issues of accessibility, affordability, and acceptability [53].

Finally, the included studies exhibited a significant lack of geographic diversity, as all 14 were conducted in either South India (*n* = 8) or North and North‐Eastern India (*n* = 8). This concentration precludes the findings from being representative of India's diverse population. Given the vast socioeconomic, cultural, and healthcare infrastructure differences across the country, the prevalence and determinants of medication adherence may vary considerably in the unrepresented western, central, and eastern regions, limiting the generalizability of the study findings.

### 4.1 Strengths and Limitations

The strengths of the study include a comprehensive SRMA conducted with a comprehensive review of the literature in a specific country with the highest diabetes burden in the world, and an explanation of the limitations in existing studies. The explicit assessment of publication bias using both “meta“ and “metafor” packages further underscores the transparency and reliability of our findings.

The present study has certain limitations. A key finding of this review is the Very Low certainty of evidence underlying the pooled adherence estimate, reflecting the systemic challenges in adherence research in India, particularly the inconsistency in methods, such as non‐reporting of the definition of adherence. Furthermore, the use of heterogeneous self‐report adherence questionnaires introduces measurement bias due to variations in recall periods and differing conceptual focuses on patient behavior versus attitudes. Also, this study was restricted to assessing medication adherence and did not evaluate patient health outcomes, such as their glycemic control. The absence of a search of gray literature may have contributed to higher publication bias.

### 4.2 Policy and Clinical Implications

The present study has certain important public health and clinical implications. The extent of medication adherence among patients with DM in India is lower than the global average, indicating both weaknesses and major opportunities for the health system. Given that enhanced medication adherence consistently improves patient health outcomes, achieving this in Indian healthcare settings would yield substantial benefits for patients, reduction of burden in terms of DALYs, with concomitant significant economic savings. Further, as multiple barriers to medication adherence were observed to be modifiable in both community and facility settings, evidence‐based interventions to improve medication adherence warrant high prioritization in Indian health settings. Considering high levels of medication non‐adherence attributable to forgetfulness, patient reminder systems leveraging low‐cost mHealth technology such as SMS reminders may have significant potential to enhance adherence [54]. Recognizing the impact of low literacy and inadequate health knowledge on non‐adherence, there's a clear need to scale up educational interventions among patients with DM in India, especially those related to “adherence counseling“ which have shown promise, especially those facilitated by nurses, pharmacists, and community health workers [55]. Simultaneously, addressing health system barriers requires monitoring and maintenance of drug refill and shielding against “financial non‐adherence“ in public health facilities, particularly at primary care levels. Prioritizing the training of healthcare providers on the consistent use of validated adherence assessment tools is essential for improving clinical management in outpatient diabetes care [56].

## 5 Conclusion

This systematic review and meta‐analysis indicate that medication non‐adherence among patients with T2DM remains a major public health concern in India. However, the certainty of this evidence is very low, and the pooled estimates should be interpreted cautiously in light of several methodological limitations identified across the included studies. A substantial proportion of studies were rated as moderate or low quality, with common risks of bias related to inadequate control of confounding, over‐reliance on self‐reported adherence measures, and limited assessment of social desirability bias. These factors are likely to have contributed to both the high heterogeneity observed and the potential overestimation of adherence levels. Furthermore, the absence of item‐wise reporting from standardized adherence scales and the lack of systematic measurement of key clinical and psychosocial factors, such as depression, disability, family support, and diabetes‐related knowledge, restrict the interpretability of pooled associations. As a result, findings related to determinants of non‐adherence should be viewed as exploratory rather than causal. Future research should prioritize methodologically robust, multi‐regional studies using validated adherence instruments with transparent item‐level reporting and appropriate adjustment for confounding factors. Addressing these sources of bias will be essential to generate more reliable estimates and to inform targeted interventions within primary care settings, particularly for modifiable factors such as health literacy, forgetfulness, and substance use.

## Author Contributions

**Saurav Basu:** conceptualization, methodology, data curation, investigation, visualization, writing – original draft, writing – review and editing, supervision. **Satyajit Kundu:** methodology, validation, investigation, visualization, writing – review and editing.

## Conflicts of Interest

The authors declare no conflicts of interest.

## Transparency Statement

The lead author Satyajit Kundu affirms that this article is an honest, accurate, and transparent account of the study being reported; that no important aspects of the study have been omitted; and that any discrepancies from the study as planned (and, if relevant, registered) have been explained.

## Supporting information

Supporting Files_R1.docx.

## Data Availability

All data are included in the article, with additional extracted data available upon reasonable request.
